# Interdialytic weight gain and low dialysate sodium concentration in patients on chronic hemodialysis: a systematic review and meta-analysis

**DOI:** 10.1007/s11255-024-03972-3

**Published:** 2024-03-06

**Authors:** Maurizio Bossola, Ilaria Mariani, Monica Sacco, Manuela Antocicco, Gilda Pepe, Enrico Di Stasio

**Affiliations:** 1 Servizio Emodialisi, Dipartimento di Scienze Mediche e Chirurgiche, Università Cattolica del Sacro Cuore, Rome, Italy; 2 Policlinico Universitario Fondazione Agostino Gemelli IRCCS, Rome, Italy; 3 Dipartimento di Medicina e Chirurgia Traslazionale, Università Cattolica del Sacro Cuore, Rome, Italy; 4 Dipartimento Scienze dell’Invecchiamento, Neurologiche, Ortopediche e della Testa-Collo, Università Cattolica del Sacro Cuore, Rome, Italy; 5 Dipartimento di Scienze Mediche e Chirurgiche, Università Cattolica del Sacro Cuore, Rome, Italy; 6 Dipartimento di Scienze Biotecnologiche di Base, Cliniche Intensivologiche e Perioperatorie, Università Cattolica del Sacro Cuore, Rome, Italy

**Keywords:** Hemodialysis, Hemodiafiltration, Sodium dialysate concentration, Interdialytic weight gain

## Abstract

**Purpose:**

The present systematic review and meta-analysis aimed at evaluating the effect of low dialysate sodium concentration on interdialytic weight gain (IDWG) in chronic hemodialysis patients.

**Methods:**

Studies were eligible for inclusion if they were English language papers published in a peer-reviewed journal and met the following inclusion criteria: (1) studies in adult patients (over 18 years of age), (2) included patients on chronic hemodialysis since at least 6 months; (3) compared standard (138–140 mmol/l) or high (> 140 mmol/l) dialysate sodium concentration with low (< 138 mmol/l) dialysate sodium concentration; (4) Included one outcome of interest: interdialytic weight gain.

Medline, PubMed, Web of Science, and the Cochrane Library were searched for the quality of reporting for each study was performed using the Quality Assessment Tool of Controlled Intervention Studies of the National Institutes of Health. The quality of reporting of each cross-over study was performed using the Revised Cochrane Risk of Bias (RoB) tool for cross-over trials as proposed by Ding et al.

**Results:**

Nineteen studies (710 patients) were included in the analysis: 15 were cross-over and 4 parallel randomized controlled studies. In cross-over studies, pooled analysis revealed that dialysate sodium concentration reduced IDWG with a pooled MD of − 0.40 kg (95% CI − 0.50 to − 0.30; *p* < 0.001). The systematic review of four parallel, randomized, studies revealed that the use of a low dialysate sodium concentration was associated with a significant reduction of the IDWG in two studies, sustained and almost significant (*p* = 0.05) reduction in one study, and not significant reduction in one study.

**Conclusion:**

Low dialysate sodium concentration reduces the IDWG in prevalent patients on chronic hemodialysis.

## Introduction

In many patients on chronic hemodialysis, interdialytic weight gain (IDWG) is greater than 4.0–4.5% of dry weight, the value recommended by the international guidelines [1]. It is well known that a high IDWG may lead to an increased risk of cardiovascular diseases and cerebrovascular diseases, with ultimately a higher risk of mortality [2–9]. Furthermore, IDWG is the cause of supplementary weekly dialysis sessions with consequent reduction of quality of life and a significant increase of economic costs.

An elevate intake of fluids and/or of foods contributes to high IDWG in patients on chronic hemodialysis. High intake of fluid is secondary to the disturbing and constant presence of thirst in such patients. Nevertheless, the adherence to fluid intake regimen is very difficult and frustrating, and rarely is successful [10–18].

The limitation of the IDWG is a challenge both for the patients and the physicians and is based on various types of interventions such the use of low dialysate sodium concentrations or patient level interventions [19].

The use of a low dialysate sodium concentration increases the removal of sodium during the dialytic session, and this leads to a reduced content of sodium in the body. As consequence, thirst is significantly reduced as well as the water intake in the interdialytic period.

Interestingly, it has been shown recently, that IDWG decreased significantly in Europe and in the USA and this was, at least in part, due to the use of a reduced sodium dialysate concentration during the dialytic session [2].

A few studies have investigated the role of a lower sodium concentration in the dialysate in reducing the IDWG in patients with chronic hemodialysis and with conflicting results [20–39]. In addition, most of these studies included a small number of patients and the number of prospective and randomized studies was limited. [20–41].

The present systematic review and meta-analysis aims to evaluate the efficacy of low dialysate sodium concentration in reducing IDWG in prevalent chronic hemodialysis patients.

## Methods

This analysis was prospectively registered on the International Prospective Register of Systematic Reviews in Health and Social Care (PROSPERO) (CRD42022359763).

### Eligibility criteria

Studies were eligible for inclusion if they were English language papers published in a peer-reviewed journal and met the following inclusion criteria: (1) primary research studies in adult patients (over 18 years of age), (2) included patients with end-stage renal disease on chronic hemodialysis since at least 6 months; (3) compared standard (138–140 mmol/l) or high (> 140 mmol/l) dialysate sodium concentration with low (< 138 mmol/l) dialysate sodium concentration; (4) Included one outcome of interest: interdialytic weight gain. We excluded studies on pediatric patients, pre-dialysis CKD patients, acute kidney injury patients, ESRD patients with other renal replacement therapy modalities, such as peritoneal dialysis and transplant.

### Information sources and search strategy

A comprehensive search to identify crossover and/or parallel randomized trials studies on effects of lower dialysate concentration on IDWG in patients on chronic hemodialysis was performed. Searches were run on January 22, 2023. The following databases were searched for relevant studies: Medline, PubMed, Web of Science, and the Cochrane Library. The search terms and mesh headings included “hemodialysis/haemodialysis” AND (“weight” OR “gain” OR “weight gain” OR “interdialytic weight gain”) AND (“salt” OR “NaCl” OR “fluid” OR “water” OR “sodium”) AND “dialysate” as the search terms. This review followed the Preferred Reporting Items for Systematic Reviews and Meta-analyses (PRISMA) reporting guideline.

### Selection process and data collection process

Two authors independently reviewed the manuscripts against the eligibility criteria and quality assessment tools**.** Two authors independently reviewed the titles and abstracts, and full texts of potential studies were retrieved for further appraisal. In case of disagreement between the two authors, a third author was referred. We also performed a manual search for eligible studies by checking the reference lists of relevant original and review articles. Conference abstracts and literature reviews were excluded. Similarly, studies not comparing standard and low sodium dialysate concentration were excluded. Any discrepancies were resolved by consensus upon discussion with another co-author. Data extraction tables (Tables 1 and 2) were compiled to record study characteristics and participant characteristics.

**Table 1:** Studies on the effect of low dialysate sodium concentration on interdialytic weight gain (IDWG): cross-over and cohort studies

	Type of study	Country	Sample size	Intervention	Duration	Outcome
Boquin., 1977	Cross-over	Canada	37	Sodium dialysate: Group 1: 130 mEq/l Group 2: 140 mEq/l	4 weeks	IDWG: 1.9 ± 0.8 kg in group1 and 2.5 ± 0.8 kg in Group 2 (*p* = 0.001)*
Ogden, 1978	Cross-over	USA	12	Sodium dialysate: Group 1: 131 mEq/l Group 2: 146 mEq/l	1 week	IDWG, 2. 2 ± 1.1 kg and 2. 6 ± 1.6 kg for the low and high dialysate groups (*p* = 0.296)*
Henrich et al.,1982	Cross-over	USA	10	Sodium dialysate: 144 vs 132 mEq/l	6 weeks	IDWG was greater with 144 mEq/l (2.3 ± 0.6) than with 132 mEq/l (1.8 ± 0.2 kg) (*p* < 0.001)*
Daugirdas et al.,1985	Cross-over	USA	7	Period 1 (4 weeks): sodium dialysate = 135 mEq/l; Period 2 (4 weeks): sodium dialysate = 143 mEq/l; Period 3 (4 weeks): sodium dialysate decreased from 160 to 133 mEq/l during each 4-h dialysis session (sodium gradient dialysate)	12 weeks	IDWG was 2.2 ± 0.9 kg in Period 1 and 2.6 ± 0.8 kg in Period 2 (*p* = 0.396) and 2.8 ± 0.7 kg in Period 3 (*p* = 0.189)*
Dominic et al., 1996	Cross-over	India	22	Shift from dialysate with 137 mEq/l sodium (conventional) to linear sodium modeling with dialysate sodium reduction from 137 to 128 mEq/l	5 weeks	IDWG was similar in low sodium dialysate HD (2.27 ± 1.21 kg) and in conventional HD (2.81 ± 0.9) (*p* = 0.10)
Farmer et al., 2000	Cross-over	UK	10	Sodium dialysate Period 1: 138–140 mEq/l Period 2: 135 mEq/l	4 weeks	IDWG: 1.7 kg or 2.6% of total body weight (range 0.9–2.4 kg) for phase 1 vs 1.8 kg or 2.8% of total body weight (range 0.8–2.4 kg) for phase 2)
De Paula et al., 2004	Cross-over	Brazil	27	Period 1: 138 mEq/l Period 2: dialysate sodium set up according to individualized sodium	6 weeks	IDWG decreased from 2.91 ± 0.87 kg to 2.29 ± 0.65 kg (*p* < 0.001)*
Thein et al., 2007	Crossover	New Zeland	42	Default change of sodium dialysate from 141 to 138 mEq/l	8 months	No change in IDWG (kg): 2.5 [2.3–2.7] vs 2.3 [2.1–2.5] (*p* = NS)**
Munoz Mendoza et al., 2011	Retrospective, observational cohort study	USA	15	Sodium dialysate Period 1: 140 mEq/l Period 2: 136 or 134 mEq/l Period 3: 140 mEq/l	36 weeks	IDWG decreased 0.6 ± 0.6 kg in Period 2 compared with Period 1 (*p* = 0.02)*
Kim et al., 2014	Cross-over	South Korea	32	Phase 1: Sodium dialysate set at 140 mEq/l Phase 2:Sodium dialysate gradually reduced from 140 to 135 mEq/l	5 months	IDWG was significantly reduced by 0.39 ± 0.38 kg (*p* < 0.01)* Phase 1: 1.9 ± 0.5 kg Phase 2: 1.5 ± 0.5 kg (*p* < 0.001)
Efitimovska-Otovic et al., 2014	Cross-over	Macedonia	92	Period 1: 138 mEq/l Period 2: dialysate sodium set up according to individualized sodium	12 weeks	IDWG decreased from 2.21 ± 0.93 to 1.87 ± 0.92 kg in Period 2 compared with Period 1 (*p* = 0.018)*
Inrig et al., 2015	Cross-over	USA	16	Low (5 mEq/l below serum sodium) versus high (5 mEq/l above serum sodium) dialysate sodium concentration	3 weeks	IDWG decreased from 2.57 ± 0.91with higher Na dialysate concentration to 2.26 ± 1.12 kg in Period 2 compared with lower Na dialysate concentration
Radhakrishnan et al., 2020	Cross-over	India	40	First phase: 12 consecutive HD sessions with dialysate sodium concentration of 140 mEq/l Second phase:, 12 consecutive HD sessions with dialysate sodium concentration set to individualized value	6 months	In the first phase, the mean IDWG was 2.64 ± 1.56 kg and 2.13 ± 0.99 kg in the second phase (*p* = 0.008)
Manji et al., 2021	Cross-over	Kenya	71	One group of patients was dialysed with g a 140 meq/l for 6 weeks, followed by 137 meq/l for the remaining 6 weeks The second group were dialysed using a Na of 137 meq/l for 6 weeks, followed by 140 meq/l for the remaining 6 weeks	12 weeks	Mean IDWG was 2.14 kg in the low dialysate sodium group (DNa 137 meq) and 2.35 in the high dialysate sodium group (DNa 140 meq) (*p* = 0.970)
Nair et al., 2021	Cross-over	India	50	Patients were dialysed for 8 sessions with 140 mEq/l dialysate and then for other 8 sessions with 136 mEq/l dialysate	5 weeks	IDWG decrease from 3.34 ± 0.9 kg to 3.11 ± 0.86 kg (*p* = 0.19) Mean dry weight at the end of standard sodium dialysate phase and low sodium dialysate phase was 53.07 ± 11.6 kg and 53 ± 11.5 kg, respectively

Data are expressed as * mean ± SD and ** median [95% CI]

**Table 2:** Studies on the effect of low dialysate sodium concentration on interdialytic weight gain (IDWG): randomized studies

	Type of study	Country	Sample size	Intervention	Duration	Outcome
Beduschi et al., 2013	Randomized, controlled	Brazil	Treated (group A) = 20 Control (group B) = 18	Sodium dialysate Group A: from 138 to 135 mEq/l Group B: maintained at 138 mEq/l	4 months	At baseline, 2 months and 4 months: IDWG (kg): 2.2 [2–2.7] and 2 [0–7-3.1] in Group A (*p* = 0.15) and 2.6 [1.7–3.5] vs 2.8 [1.4–3.2] in Group B (*p* = 0.114)**
Akdag et al., 2015	Randomized, controlled	Turkey	Treated (group A) = 22 Control (group B) = 24	Sodium dialysate: Group A: 137 mEq/l Group B: 140 mEq/l	6 months	IDWG at baseline and 6 months: Group A: 2.2 ± 0.9 kg and 1.6 ± 0.5 kg (*p* < 0.001) Group B: 2.3 ± 0.8 kg vs 2.1 ± 0.7 kg (p > 0.05)*
Liu et al., 2016	Randomized, controlled	China	Treated (group A) = 28 Control (group B) = 29	Intervention group: sodium dialysate reduced from 138 to 136 mEq/l Control group: sodium dialysate maintained at 138 mEq/l	12 months	IDWG at baseline and 12 months: Group A: 3.3 ± 0.7 kg and 2.8 ± 0.6 kg (p < 0.05) Group B: 3.1 ± 0.8 kg vs 3.0 ± 1 kg (*p* > 0.05)*
Marshall et al., 2020	Randomized, controlled	New Zeland	99	Intervention group: sodium dialysate reduced to 135 mEq/l Control group: sodium dialysate maintained at 140 mEq/l	12 months	IDWG was reduced of − 0.56 kg [− 0.86 to − 0.27] in the treatment group at 12 months (*p* = 0.05)

Data are expressed as * mean ± SD and ** median [95% CI]

### Data items

The outcome for which data was sought was IDWG.

### Study risk of bias assessment

The quality of reporting for each study was performed by two researchers (GP, MA) using the Quality Assessment Tool of Controlled Intervention Studies of the National Institutes of Health [42].

The quality of reporting of each cross-over study was performed by two researchers (GP, MA) using the Revised Cochrane Risk of Bias (RoB) tool for cross-over trials as proposed by Ding et al. [43].

### Statistical analysis / synthesis methods

The primary outcome was the mean IDWG and compared in terms of the mean difference (MD).

Statistical heterogeneity among studies was assessed with Cochran’s *Q* and quantified with Higgins *I*^2^ statistic [44–48]. Because of low heterogeneity (*I*^2^ = 0%), data were analyzed using a fixed effect (Mantel–Haenszel method) model approach [45].

Publication bias was assessed graphically using funnel plots. Statistical analysis was performed using the Statistical Package for Social Science (SPSS 22.0; SPSS Inc, Chicago, IL, United States) and Microsoft Excel (Version 16.45).

### Certainty assessment

Overall evidence was qualified using Grading of Recommendations Assessment, Development, and Evaluation (GRADE) working group guidelines for both cross-over and parallel-group observational studies. Results of the quality of evidence criteria are reported in Tables 3 and 4.

**Table 3:** Quality analysis of cross-over studies on the effect of low dialysate sodium concentration on interdialytic weight gain

Authors	1	2	3	4	5	6	7	8	9	Overall
Boquin., 1977	Low	Unclear	Unclear	Low	Unclear	Low	Low	Low	Low	Low
Ogden, 1978	Low	Unclear	Unclear	Low	Unclear	Low	Low	Low	Low	Low
Henrich et al.,1982	Low	Low	Unclear	Low	Unclear	Low	Low	Low	Low	Low
Daugirdas et al.,1985	Low	High	Unclear	Low	Unclear	Low	Low	Low	Low	Low
Dominic et al., 1996	Low	High	Unclear	Low	Unclear	Low	Low	Low	Low	Low
Farmer et al., 2000	Low	Low	Unclear	Low	Unclear	High	Low	Low	Low	Low
De Paula et al., 2004	Low	High	Unclear	Low	High	High	Low	Low	Low	Some concerns
Thein et al., 2007	Low	Unclear	Unclear	Low	Unclear	High	Low	Low	Low	Some concerns
Munoz Mendoza et al., 2011	Low	Low	Unclear	Low	Low	Low	Low	Low	Low	Low
Kim et al., 2014	Low	Unclear	Unclear	Low	Unclear	Unclear	Low	Low	Low	Some concerns
Efitimovska-Otovic et al., 2014	Low	Unclear	Unclear	Low	Unclear	Unclear	Low	Low	Low	Some concerns
Inrig et al., 2015	Low	Low	Unclear	Low	Unclear	Low	Low	Low	Low	Low
Radhakrishnan et al., 2020	Low	High	Unclear	Low	Unclear	Low	Low	Low	Low	Some concerns
Manji et al., 2021	Low	Low	Unclear	Low	Unclear	Unclear	Low	Low	Low	Low
Nair et al., 2021	Low	High	Unclear	Low	Unclear	Low	Low	Low	Low	Low

Legend: 1. appropriate cross-over design; 2. the randomized order of receiving treatment; 3. carry-over effects; 4. unbiased data; 5. allocation concealment; 6. blinding; 7. incomplete outcome data; 8. selective outcome reporting; 9. other biases

**Table 4:** Quality analysis of prospective, randomized, controlled studies on the effect of low dialysate sodium concentration on interdialytic weight gain

Authors	1	2	3	4	5	6	7	8	9	10	11	12	13	14	Overall score
Beduschi et al., 2013	Y	NR	NR	N	NR	Y	NR	NR	Y	Y	Y	NR	Y	Y	7
Akdag et al., 2015	Y	Y	Y	N	NR	Y	Y	Y	Y	Y	Y	Y	Y	Y	12
Liu et al., 2016	Y	Y	Y	N	NR	Y	Y	Y	Y	Y	Y	Y	Y	Y	12
Marshall et al., 2020	Y	Y	Y	N	NR	Y	Y	Y	Y	Y	Y	Y	Y	Y	12

Y = Yes; N = No; NA = Not applicable; NR = Not reported

Legend: 1. Was the study described as randomized, a randomized trial, a randomized clinical trial, or an RCT? 2. Was the method of randomization adequate (i.e., use of randomly generated assignment)? 3. Was the treatment allocation concealed (so that assignments could not be predicted)? 4. Were study participants and providers blinded to treatment group assignment? 5. Were the people assessing the outcomes blinded to the participants’ group assignments? 6. Were the groups similar at baseline on important characteristics that could affect outcomes (e.g., demographics, risk factors, co-morbid conditions)? 7. Was the overall drop-out rate from the study at endpoint 20% or lower of the number allocated to treatment? 8. Was the differential drop-out rate (between treatment groups) at endpoint 15 percentage points or lower? 9. Was there high adherence to the intervention protocols for each treatment group? 10. Were other interventions avoided or similar in the groups (e.g., similar background treatments)? 11. Were outcomes assessed using valid and reliable measures, implemented consistently across all study participants? 12. Did the authors report that the sample size was sufficiently large to be able to detect a difference in the main outcome between groups with at least 80% power? 13. Were outcomes reported or subgroups analyzed prespecified (i.e., identified before analyses were conducted)? 14. Were all randomized participants analyzed in the group to which they were originally assigned, i.e., did they use an intention-to-treat analysis?

## Results

### Search results (study selection)

**Fig. 1:**
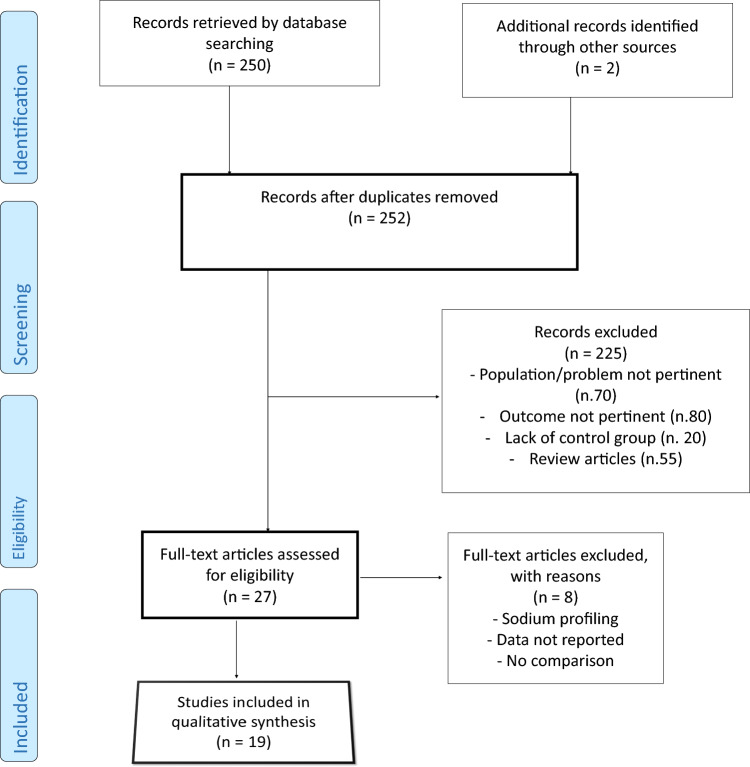
Preferred reporting items for systematic reviews and meta-analyses (PRISMA) flowchart of our analysis

A total of 1240 publications were identified via electronic databases. After screening the titles, abstracts, and full texts, 19 studies that met the inclusion criteria were included for analysis. The PRISMA flowchart is shown in Fig. 1. Of the 19 included studies, 15 were cross-over studies and 4 were parallel groups studies. We divided the analysis into two sections: (1) comparison of standard and low dialysate sodium concentration through crossover prospective studies comprising 15 studies; (2) comparison of standard and low dialysate sodium concentration through prospective randomized parallel studies, comprising 4 studies.

### Study characteristics

#### Comparison of standard and low sodium dialysate concentration through crossover prospective studies

Overall, 483 patients were included. The number of patients in each individual study ranged from 7 to 92. The used dialysate sodium concentrations (mEq/l) varied largely: 138 vs 141 in one study, 135 vs 140 in two studies, 137 vs 140 in one study, 130 vs 140 in one study, 136 vs 140 in two studies, 135 vs 138 in one study, 135 vs 143 in one study, 132 vs 144 in one study, 134 vs 144 in one study, 131 vs 146 in one study, 138 vs individualized sodium concentration in two studies, 140 vs individualized sodium concentration in one study. Description of the included studies is presented in Table 1. The cross-over studies differed for some variables (age: ranged from 45.6 to 67 years; sex distribution [male%]: ranged 48.1–93.7; sample size: ranged from 10 to 92 patients) but not for others (use of HDF absent in all studies; use of diuretics absent in all studies but one; wash out absent in all studies but one; report of renal residual function absent in all studies but two; use of dietary counseling absent in all studies; data on hospitalization and mortality absent in all studies).

#### Comparison of standard and low sodium dialysate concentration through prospective randomized parallel studies

Overall, 247 patients were included. The number of patients in each individual study ranged from 38 to 99. The used sodium dialysate concentrations (mEq/l) were: 135 vs 138 in one study, 136 vs 138 in one study, 135 vs 140 in one study, and 137 vs 140 in one study. Description of the included studies is presented in Table 2. The randomized, parallel studies differed for some variables (age: ranged from 44 to 65 years; sex distribution [male%]: ranged from 45.6 to 67.6; sample size: ranged from 38 to 99 patients) but not for others (use of HDF absent in all studies; use of diuretics absent in all studies; report of renal residual function absent in three studies and present in one; use of dietary counseling absent in three studies and present in one; data on hospitalization and mortality absent in all studies).

### Efficacy of low dialysate sodium concentration on IDWG

#### Comparison of standard and low dialysate sodium concentration through prospective crossover studies

**Fig. 2:**
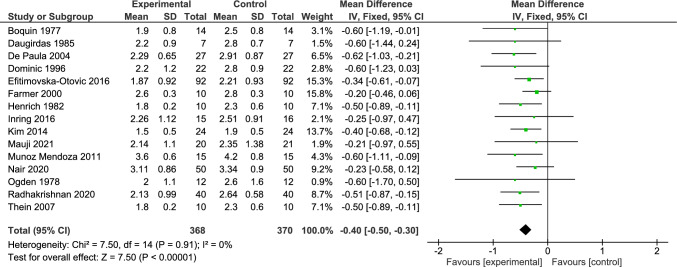
Forest plot of comparison between low dialysate sodium concentration and high dialysate sodium concentration about interdialytic weight gain (kg) in crossover studies

**Fig. 3:**
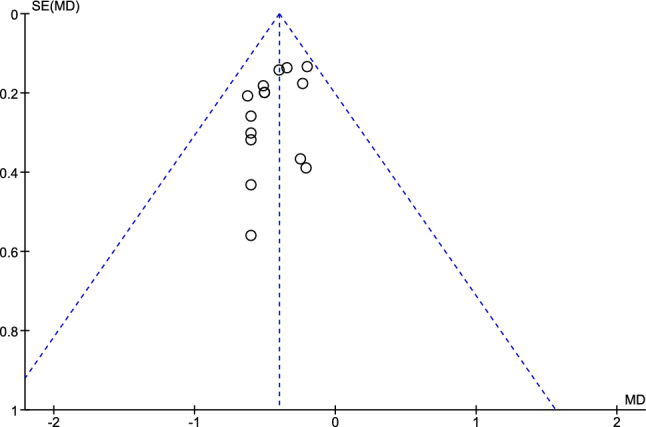
Funnel plot for interdialytic weight gain in crossover studies

As shown in Fig. 2, compared to neutral or high dialysate sodium concentration, low dialysate sodium concentration reduced IDWG with a pooled MD of − 0.40 kg (95% CI − 0.50 to − 0.30; *p* < 0.001). As no significant heterogeneity was observed (*p* = 0.91 and *I*^2^ = 0%), the pooled analysis was performed using a fixed-effect model. Funnel plots were generated to assess publication bias in the included studies. As shown in Fig. 3, no obvious asymmetry, indicating no clear evidence of publication bias, was observed.

#### Comparison of standard and low dialysate sodium concentration through prospective randomized parallel studies

A two-group, parallel-design study randomized 99 patients to 1:1 to either intervention (lower dialysate sodium concentration at 135 mmol/l) or control (conventional dialysate [Na+] at 140 mmol/l) group. The primary outcome was left ventricular mass index. The secondary outcomes were IDWG, intradialytic blood pressure, xerostomia, and thirst. The intervention resulted in a sustained, almost significant (*p* = 0.05) decrease of IDWG at 3 (− 0.47 [− 0.80 to − 0.15]), 6 (− 0.56 [− 0.90 to − 0.22]), 9 (− 0.90 [− 1.26 to − 0.54]), and 12 months (− 0.57 [− 0.86 to − 0.27]) [36]. In the study of Liu et al. [37], after 1-month period of dialysis with standard dialysate sodium concentration at 138 mmol/l (baseline), the dialysate sodium was switched to 136 mmol/l in the intervention group (28 patients), whereas remained at 138 mmol/l in the control group (29 patients). Each patient was followed up over a 12-month period. IDWG at baseline and 12 months was in the intervention group 3.3 ± 0.7 kg and 2.8 ± 0.6 kg (*p* < 0.05) and, in the control group, 3.1 ± 0.8 kg and 3.0 ± 1 kg (*p* > 0.05). Akdag et al. [38] randomized 46 patients, dialyzed with 140 dialysate sodium concentration, into a control group (24 patients) that maintained the dialysate 140 mmol sodium concentration and a treatment group in which the dialysate sodium concentration was reduced to 137 mmol/l. IDWG at baseline and 6 months was, in the intervention group, 2.2 ± 0.9 kg and 1.6 ± 0.5 kg (*p* < 0.001) and, in the control group, 2.3 ± 0.8 kg and 2.1 ± 0.7 kg (*p* > 0.05). In the study of Beduschi et al. [39], 38 patients were randomized to a control group (18 patients) with dialysate sodium concentration of 138 mmol/l or an intervention group (20 patients) with dialysate sodium concentration of 135 mmol/l. At baseline, 2 months and 4 months, the IDWG (kg) was 2.6 [1.7–3.5], 2.3 [1.84–2.9], and 2.8 [1.4–3.2] in the control group (*p* = 0.114) and 2.2 [2–2.7], 2 [0.75–3.03] in the treatment group (*p* = 0.11).

### Risk of bias in studies

As shown in Table 3, cross-over design was appropriate in all studies. Five out of 15 crossover studies randomly allocated participants. Carry-over effects were not reported in any of the studies. Allocation of concealment was not reported in any crossover studies. Blinding of participants or study personnel was present in 9 studies, absent in 3, and indeterminate in 3. Data for every period were provided in every study. Relevant information about the completeness of outcome data was provided in every study. All the outcomes were fully reported in every study. All studies were apparently free of other problems. The overall rating was “low risk of bias” in 10 studies and “some concerns of bias” in 5 studies.

Table 4 shows that the overall score of randomized, controlled studies comparing standard and low sodium dialysate concentration ranged from 7 to 12, being 12 in three studies and 7 in one study.

Using GRADE, the certainty of the meta-analytic evidence (the extent of confidence the estimate of effect is correct) was considered high certainty. We did not strongly suspect publication bias in the studies eligible for the meta-analysis. We judged the evidence to have no serious indirectness, because despite variability, studies compared interventions to hemodialysis populations, and all studies used a direct measure of IDWG as the primary outcome. Considering that the sample size in the meta-analysis (*n* = 483) meets the optimal information size criterion, we did not downgrade due to imprecision.

### Effect of low dialysate sodium concentration on intradialytic hypotensive events

In eleven studies, the number of intradialytic hypotensive events per total number of dialytic sessions has been reported. In most of these studies (9 out of 11), the frequency of intradialytic hypotensive events did not differ significantly between treatments performed with low and high sodium dialysate concentrations (Table 5). In the study of Marshall et al., lower sodium dialysate concentration resulted in a progressive increase of the frequency of intradialytic hypotensive events, with risk (OR) of 1.5 [0.2–10.2] at 3 months, 3.44 [0.5–23.6] at 9 months and 3.6 [0.5–28.8] at 12 months.

**Table 5:** Frequency of intradialytic hypotensive events: comparison of low and high sodium dialysate concentrations

	Number of patients	Low sodium dialysate	High sodium dialysate	*p*
Henrich et al., 1982	10	0%	0%	1.000
Farmer et al., 2000	10	0%	0%	1.000
De Paula et a, 2004	27	2.4%	9.4%	**0.001**
Thein et al., 2007	42	0.6%	1.3%	0.264
Munoz Mendoza et al., 2011	15	3.8%	5.5%	0.620
Beduschi et al., 2013	38	1.56%	0.69%	0.122
Inrig et al., 2016	16	4.4%	0%	0.231
Eftimovska-Otovic et al., 2016	92	0.09%	0%	0.333
Radhakrishnan et al., 2020	40	10%	18.3%	** < 0.001**
Manji et al., 2021	71	0.27%	0.52%	1.000
Nair et al., 2021	48	1.04%	1.3%	1.000

## Discussion

The meta-analysis of 13 crossover randomized studies shows that the use of a low dialysate sodium concentration reduces the IDWG in prevalent patients on chronic hemodialysis, with a pooled MD of − 0.42 kg. In addition, the present systematic review of four parallel, randomized, controlled studies reveals that the use of a low dialysate sodium concentration is associated with a significant reduction of the IDWG in two studies, sustained and almost significant (*p* = 0.05) in one study, and not significant in the fourth one.

It could be questioned that the − 0.40 kg reduction observed in the pooled analysis may represent an effect that can be considered as being clinically small. Indeed, it seems that, when compared with other studies [2, 36], such reduction in considerable and deserve attention. In the Dialysis Outcomes and Practice Patterns Study (DOPPS), the IDWG reduction, during a 10-year period of observation, was − 0.29 kg and − 0.25 kg, in the USA/Canada and in Europe, respectively [2]. Surprisingly, two recent systematic reviews have led to conflicting results [49, 50]. Basile et al. reported that, in most studies included in their review, patients on higher sodium dialysate had a significantly higher IDWG and that three interventional studies found no substantial differences in such parameter while in one study a reduction in total body weight in both patients undergoing low (135 mmol/l) or high (140 mmol/l) dialysate sodium concentration, but the difference between these two groups did not attain statistical significance [49]. The results of the study of Dunlop et al. demonstrated that low dialysate reduced IDWG compared to neutral or high dialysate (10 studies, 352 participants: MD − 0.35 kg, 95% CI − 0.51 to − 0.18), but the results were considered by the authors themselves as being clinically small [50].

However, it has been argued that the use of a low dialysate sodium concentration may be, in some cases, associated with adverse events such as intra-dialytic hypotension, as consequence of an intradialytic hemodynamic instability [36, 49].

Interestingly, the present systematic review shows that in most studies, the frequency of intradialytic hypotensive events did not differ significantly between patients receiving hemodialysis with low or high sodium dialysate concentration [22, 26, 27, 30, 32, 34, 35, 39]. The study of Marshall et al. only reported that hypotensive events were more frequent with lower sodium dialysate concentration [36]. The present review has some limitations. First, the length of the studies was extremely variously ranging from one week to twelve months. In this regard, it remains essentially unknown the mid-term and long-term effect of the use of low sodium dialysate concentration. Second, the “low” dialysate sodium concentrations used in the 20 studies were also extremely various. Consequently, it is impossible to define an “ideal” dialysate sodium concentration to be used in the clinical practice to reduce the IDWG. Third, studies were characterized by high complexity and heterogeneity.

In conclusion, the present systematic review and meta-analysis suggests that the use of a low sodium concentration in the dialysate is associated with a statistically significant reduction of the IDWG in patients on chronic hemodialysis without a significant increase of intradialytic hypotensive events. Given the high complexity and diversity of the studies, it is amenable that, a prospective, randomized study with an adequate sample size and sex distribution, definition of residual renal function and use of diuretics, use of dietary counseling during the study, definition of the difference between prescribed and serum sodium concentration will be performed in the next future.
